# Bing-Neel Syndrome with Detectable MYD88 L265P Gene Mutation as a Late Relapse Following Autologous Hematopoietic Stem Cell Transplantation for Waldenström’s Macroglobulinemia

**DOI:** 10.4274/tjh.2016.0452

**Published:** 2017-06-01

**Authors:** Anna J. Kopińska, Grzegorz Helbig, Anna Koclęga, Sławomira Kyrcz-Krzemień

**Affiliations:** 1 Silesia University Faculty of Medicine in Katowice, Department of Hematology and Bone Marrow Transplantation, Katowice, Poland

**Keywords:** Bing-Neel syndrome, Waldenström’s macroglobulinemia, Central nervous system, MYD88 L265P mutation, Cerebrospinal fluid, Leukemia

## To The Editor,

Waldenström’s macroglobulinemia (WM) affects a proportion of patients diagnosed with lymphoplasmacytic lymphoma with bone marrow involvement and Immunoglobulin M (IgM) monoclonal gammopathy of any concentration [1]. The direct central nervous system (CNS) infiltration by malignant lymphoid cells is a rare complication of WM known as Bing-Neel syndrome (BNS) [2]. The *MYD88* L265P point mutation is detected in about 90% of WM patients and may serve as a marker in distinguishing WM from other lymphomas [3]. It is noteworthy that this mutation has recently been detected in the cerebrospinal fluid (CSF) of patients with BNS [4].

A 67-year-old female with an 8-year history of WM presented with quadriplegia. At initial diagnosis her bone marrow was infiltrated by plasmacytoid lymphocytes with expression of typical WM surface markers (CD5^+^, CD10^+^, CD19^+^, CD20^+^, CD22^+^, CD23^-^, CD43^+^, CD79a^+^, CD200^+^, kappa^+^, lambda^-^). A monoclonal spike at 23 g/L was demonstrated in serum electrophoresis (SPE) and serum immunofixation (IFE) detected IgM kappa protein. She received cladribine with cyclophosphamide. As a result, she achieved complete response and was autotransplanted. Seven years later, the patient started complaining of disturbed gait. Neurological examination showed quadriplegia and ataxia. Magnetic resonance imaging (MRI) of the brain was not performed due to the presence of a pacemaker. Bone marrow aspirate was free of WM. CSF analysis identified lymphoplasmocytoid cells with the WM immunophenotype. A monoclonal spike of IgM kappa was demonstrated in CSF but not in SPE/IFE. The *MYD88* L265P gene mutation was found in the CSF, but not in the marrow. She received intrathecal therapy with intravenous high-dose methotrexate and ifosfamide. While still on therapy, she progressed 2 months later. A complete blood count revealed extremely elevated white blood cells (356x10^9^/L). Blood and marrow smears revealed >90% plasmacytoid lymphocytes with WM surface markers. The *MYD88* L265P mutation was detected in her blood. SPE and IPE confirmed the presence of M-protein at a high level (33 g/L). She received palliative care.

BNS is a rare complication of WM and may have different clinical features. The diagnosis often remains challenging and includes the combination of CSF cytology and flow cytometry, MRI, and the detection of the *MYD88* L265P mutation. A consensus on the diagnostic algorithm, recommended treatment, and response criteria was published recently [5]. This novel mutation may be helpful in monitoring minimal residual disease in BNS after treatment; however, this claim is based on a single report [6]. Of note is that the presence of the *MYD88* mutation in the CSF is not synonymous with BNS. Its detection may result from blood contamination as small lymphocytes cross the blood-brain barrier [7]. Moreover, other CNS lymphomas may harbor this mutation and therefore it is not specific [8]. The choice of treatment strategy for patients with BNS is still a matter of debate. The majority of patients were treated with systemic chemotherapy combined with intrathecal chemotherapy [9]. It was recently suggested that treatment with ibrutinib may be successful in patients with BNS [10]. Long-term treatment for WM may result in the development of secondary hematological malignancies [1]. However, a leukemic transformation of WM has not been reported so far.
